# Equitable access to essential medicines to treat neglected tropical diseases: Latest developments from the World Health Organization (WHO)

**DOI:** 10.1371/journal.pntd.0013854

**Published:** 2025-12-23

**Authors:** Fanan Fattah, Richard Lester, Khumbo Kalua, Brett Sharp, Kishor M. Wasan

**Affiliations:** 1 The Neglected Global Diseases Initiative, The University of British Columbia, Vancouver, British Columbia, Canada; 2 Department of Medicine, Division of Infectious Diseases, The University of British Columbia, Vancouver, British Columbia, Canada; 3 School of Population and Public Health, Faculty of Medicine, The University of British Columbia, Vancouver, British Columbia, Canada; 4 University-Industry Liaison Office, The University of British Columbia, Vancouver, British Columbia, Canada; 5 Department of Urologic Sciences, Faculty of Medicine, The University of British Columbia, Vancouver, British Columbia, Canada; University of Massachusetts Amherst, UNITED STATES OF AMERICA

## Abstract

In July 2023, the World Health Organization (WHO) released the 23rd Model List of Essential Medicines (EML) for adults and the 9th Model List of EML for children. These lists serve as global references for essential medicines that should be universally available, accessible, and affordable. This editorial explores recent updates to the WHO EML lists, with a particular focus on medicines addressing neglected tropical diseases (NTDs). We discuss the persistent barriers to equitable access, including economic, regulatory, and logistical challenges; highlight global initiatives aimed at improving accessibility; and propose strategies to bridge the gap in essential medicine access. We emphasize the need for international collaboration, increased funding for research institutions, pharmaceutical companies, and global health initiatives to support the development of novel treatments for NTDs, and innovative distribution approaches to ensure these medications reach underserved populations.

## 1. Introduction

Essential medicines are chosen according to a population’s health priorities. Selection is made based on disease prevalence, evidence for efficacy and safety, and cost-effectiveness—criteria established by the World Health Organization (WHO) and reaffirmed in 2024 [1,2]. These same criteria were applied to the 23rd Model List of Essential Medicines (EML) in 2023, which this editorial examines. Since its first publication in 1977, the EML has guided national medicine policies and improved the availability of life-saving medicines to all who need them [3], yet around 2 billion people globally still lack access to essential medicines, particularly in low- and middle-income countries (LMICs) [1–4]. Recent analyses confirm that affordability challenges remain pressing even in 2025, with essential generics still unaffordable for many households [5].

Neglected tropical diseases (NTDs) are ancient illnesses of poverty that impose a devastating human, social, and economic burden on over 1 billion people annually [2], primarily in tropical and subtropical regions among vulnerable, marginalized populations. Over 90% of NTDs affect LMICs, thriving in environments marked by inadequate sanitation and weak healthcare infrastructure [6]. NTDs include, but are not limited to, lymphatic filariasis, trachoma, onchocerciasis, leishmaniasis, and schistosomiasis. Despite their significant impact, NTDs remain underfunded in both research and pharmaceutical industry investments compared to other global diseases. Although NTDs represented nearly 20% of the infectious disease burden in 2022, they still accounted for less than 0.5% of global health Research and Development (R&D) funding [7].

The 23rd Model List of Essential Medicines of the WHO (2023) is significant for its increased emphasis on infectious diseases, antimicrobial stewardship, and the equitable medicine distribution. Nevertheless, the primary obstacles to accessing essential medicines in the LMICs remain. This editorial will highlight some of these challenges and illustrate how real-world evidence can be used to address them in the context of policymaking. Although malaria is not classified by the WHO as a NTD [8], it disproportionately affects underserved and vulnerable populations in many LMICs and faces similar barriers to equitable medicine access. Therefore, in this editorial, malaria is included in certain sections as a comparative example to highlight overlapping challenges and potential shared solutions.

## 2. Background: The WHO Model List of Essential Medicines

The WHO Model List of Essential Medicines (EML) was first introduced in 1977 to assist countries in identifying those essential and cost-effective medicines that should be made available to the population [1]. The list has become a global reference. Updated every two years, the list now informs national essential medicine policies in more than 110 countries [3].

The WHO EML criteria for selecting medicines are:

1. Disease prevalence: These are disorders that are most likely to affect the population locally or globally.
2. Efficacy and safety: Evidence from randomized controlled trials and meta-analysis should support the use of the drug and prove its efficacy, safety, and tolerance.
3. Comparative cost-effectiveness: They are the most effective and cost-effective strategies that countries should consider implementing to achieve value for money and reduce the financial burden on patients and health systems.

The WHO EML is further divided into two lists:

- The Core List which contains the medicines that are most important to address the primary global health issues.
- The Complementary List which contains medicines that need special equipment or blood monitoring to avoid severe adverse effects but are essential in the management of specific diseases [1].

By serving as a global reference, the EML guides procurement, supply chain planning, and policymaking at the national level [1]. It also plays a role in supporting universal health coverage by identifying treatments that should be accessible, affordable, and available to all populations [4].

### 2.1. Updates to the 2023 WHO Essential Medicines Listing

The 55 new medicines were recommended for inclusion in the 23rd WHO Model List (2023), and 9 existing drugs had their indications expanded. This update focuses on infectious diseases, antimicrobial resistance (AMR), and affordability [1].

- New Antimicrobial Stewardship Measures:
  - The updated EML classifies antibiotics into three groups (Access, Watch, and Reserve) to fight against the rising problem of AMR.
  - New guidelines for the appropriate use of antibiotics to help prevent the emergence of resistant bacteria, a major threat to healthcare systems, especially in LMICs [3].
- Malaria is included here to highlight similarities in the access issues that other diseases affecting neglected populations face, even though it is not an NTD according to WHO’s current classification. Strategies relevant to NTDs can be informed by insights from access to malaria treatment. Improved Availability of Malaria Treatments:
  - The current list of recommendations for the treatment of malaria is still based on artemisinin-based combination therapies (ACTs), with new formulations that are supposed to be more effective in the context of emerging resistance [1].
- New formulations of pediatric NTD drugs:
  - The new list identifies child-friendly drug formulations for the treatment of parasitic infections, leishmaniasis, and schistosomiasis, which are important in enhancing child survival in the NTD endemic areas [8].
- More emphasis on affordability and universal health coverage (UHC):
  - The WHO is advised to employ price negotiations and the pooled procurement as a way of ensuring that the newly included essential medicines are within the reach of the LMICs [9].

#### 2.1.1. NTD-Specific Implications of the 2023 EML.

The 2023 WHO EML includes several additions and revisions that directly impact the prevention and treatment of NTDs. Primarily, the inclusion of pediatric formulations for leishmaniasis and schistosomiasis addresses long-standing gaps in child-appropriate dosing and administration, a key barrier in endemic regions [1,8]. Updated recommendations for antiparasitic medicines enhance treatment efficacy and may help mitigate resistance development [1]. Although malaria is not classified as an NTD, the addition of newer artemisinin-based combination therapies offers lessons in scaling up access to essential medicines for other diseases affecting neglected populations [8]. These updates, while important, highlight remaining gaps; for example, the absence of certain NTD treatments from the core list, limited guidance on affordable procurement mechanisms for LMICs, and the need for stronger links between the EML and national procurement systems [1,9].

The 2023 updates are consistent with the WHO’s efforts to address issues of equity in access to medicines on a global level. However, policy changes and more funding are required for real-world adoption.

### 2.2. NTDs and Their Worldwide Burden

More than one billion people are affected by NTDs, mainly in Africa, South Asia, and Latin America [2]. These diseases hold and maintain people in the poverty cycle through long-term health impairment, disability, and reduced economic productivity. Some examples are:

- Schistosomiasis: It is estimated that 251 million people have been affected globally, but access to treatment is not equitable [4]
- Leishmaniasis: It is estimated that between 700,000 and 1 million new cases are reported annually, less than 10% of patients in endemic areas get the treatment on time because of unavailability and high cost of the treatment [7].
- Lymphatic Filariasis: It is a major cause of permanent disability, and its prevalence is highest among disadvantaged populations.

However, there has been less investment in research and development for NTDs than for diseases that are more common in high-income countries, resulting in the treatment gap and slow development of new treatments [6].

## 3. Barriers to equitable access

Approximately 2 billion people, especially in LMICs, are still faced with great challenges in obtaining the essential medicines as data shows that over 50% of public health facilities in LMICs experience regular stock-outs [10]. These barriers are a result of economic factors, policy barriers, weak health systems, and pharmaceutical industry factors [4,11].

Despite persisting challenges, global access to essential medicines did not stop improving. WHO reports that global access to essential medicines rose from 50% in 2015 to 65% in 2022 [1]. The upward trend in access demonstrates successful worldwide initiatives, but LMICs continue to face substantial access challenges.

### 3.1. Economic constraint

The high cost and low health care financing in LMICs is a major factor that limits the access to essential medicines for NTDs. The inability of most national health systems to afford essential medicines has led to stockouts or mimicking donor-dependent programmes [12–15]. Recent WHO reporting and 2025 analyses reaffirm that affordability gaps persist despite progress [5].

### 3.2. Regulatory and logistical constraints

Access to medicines in LMICs is frequently hindered by regulatory and logistical barriers. Lengthy drug approval processes delay market entry, while weak procurement and distribution systems contribute to recurring stock-outs of essential medicines. Inadequate quality control and limited regulatory oversight also increase the prevalence of counterfeit or substandard products. Together, these challenges create systemic barriers that prevent timely and equitable access to essential medicines in LMICs [9,16,17].

### 3.3. Poor engagement from the pharmaceutical industry

The lack of profits in the market for NTDs removes incentives for pharmaceutical companies to conduct research and development of new treatments. This market failure can be addressed by public–private partnerships (PPPs), subsidies, and advance market commitments that reduce the financial risks and ensure demand [10]. Funding, tax incentives, and volume guarantees are some of the mechanisms that provide funding and make NTD treatments more attractive to pharmaceutical investors [18,19].

#### 3.3.1. Barrier: Lack of demand certainty (advanced purchase commitments).

Uncertainty of demand discourages pharmaceutical companies from investing in NTD treatments. Without predictable markets, R&D for low-profit diseases remains unattractive. The financing tool known as Advanced Purchase Commitments (APCs) helps minimize financial risks that come from developing treatments for NTDs. The guaranteed market through APCs gives pharmaceutical companies the necessary certainty to conduct R&D for diseases that generate limited profits. The Global Fund together with Gavi, the Vaccine Alliance (Gavi) has implemented APCs to promote vaccine development for malaria and rotavirus while similar models should be used for NTDs [10]. Similar models, if applied to NTDs, could help overcome this barrier and stimulate innovation [18,19]

#### 3.3.2. Barrier: Unaffordable pricing models (tiered pricing).

Pricing remains a fundamental barrier to equitable access for NTD medicines. Pharmaceutical companies implement tiered pricing structures which enable them to establish varying medication costs based on the income level of each purchasing nation. While the HIV/AIDS sector demonstrates successful implementation of this pricing model through antiretroviral drugs that maintain affordability for low-income nations, NTD medicines have not benefited from similar arrangements. The absence of transparent fair pricing model and mechanisms creates a barrier for LMICs that must fund NTD programs largely without donor subsidies. Without adaptation of tiered pricing to NTD medicines, affordability remains a critical obstacle to equitable access [5,12–14]

#### 3.3.3. Barrier: Lack of R&D incentives (tax credits).

Pharmaceutical companies often lack financial incentives to pursue R&D for NTDs, as these conditions primarily affect low-income populations with limited market potential. Tax credits serve as incentives that can motivate pharmaceutical companies to invest in NTD drug development. Governments could provide tax reductions to pharmaceutical companies that dedicate specific parts of their R&D funding to neglected diseases. This approach would help ease the financial burden on companies while enhancing their interest in developing treatments for NTDs.

A relevant example is the Orphan Drug Tax Credit in the United States, which successfully stimulated investment in therapies for rare diseases by reducing the effective cost of development [12]. Analyses of the neglected disease R&D landscape highlight persistent financing gaps and underscore the need for targeted incentives to overcome the innovation deficit [18,19]. More recently, frameworks such as the UBC Global Access Principles have demonstrated how aligning innovation incentives with equitable access goals can further ensure that new medicines benefit populations in LMICs [20]. Adapting such tax-based incentives for NTDs could help fill critical innovation gaps and align private sector efforts with public health needs.

## 4. Global initiatives and the role of real-world evidence

NTDs are a subset of infectious diseases that account for 19% of global disease burden, yet they have obtained less than 0.5% of global health funding and development funding during the last decade—far less than HIV/AIDS, tuberculosis, or malaria [4,7].

WHO’s initiatives and rising use of real-world evidence attempt to address these deficiencies by providing new approaches to enhance the availability, affordability, and distribution of these potentially life-saving drugs. Notwithstanding these efforts and the increasing momentum in the global fight against NTDs by international health organizations, governments, pharmaceutical companies, and non-governmental organizations (NGOs), the challenge of equitable access to essential medicines persists.

### 4.1. WHO initiatives

WHO is central in setting up global standards as well as frameworks for action to combat NTDs. Accelerating programmatic action, increasing cross-sectoral collaboration, and strengthening country ownership are the three key strategic pillars of the WHO’s activities in this regard based on the Global Strategy for Control of Neglected Tropical Diseases (2021–2030) [21]. Some of the components are:

#### 4.1.1. Mass drug administration (MDA).

In countries with high prevalence of certain diseases including lymphatic filariasis, onchocerciasis, and schistosomiasis, the World Health Organization has recommended preventive chemotherapy and has initiated Mass Drug Administration (MDA) campaigns. In this regard, the 2021 MDA campaign covered more than 800 million people in sub-Saharan Africa and South Asia [1] with the assistance of the WHO.

#### 4.1.2. Enhancing the procurement systems of pharmaceuticals.

The WHO works with organizations like the Global Drug Facility and the Medicines Patent Pool to ensure the availability and affordability of essential medicines that include antimalarials, antiparasitic, and tuberculosis drugs [22]. By voluntary licensing agreements, generic competition, and pooled procurement mechanisms, these partnerships have brought down prices and improved access to medicine in LMICs. For instance, licensing of intellectual property has helped in the affordable procurement of essential medicines in LMICs, resulting in reduced costs and increased treatment coverage [23].

#### 4.1.3. Partnerships with NGOs and other international organizations.

Other global health organizations, including Drugs for Neglected Diseases Initiative (DNDi), Gavi, and the Bill & Melinda Gates Foundation, have also been involved in financing and delivering essential medicines to WHO. DNDi has provided affordable treatments for leishmaniasis, sleeping sickness, and Chagas disease and more than 2.4 million people have benefited from the life-saving drugs [7]. Especially, the Gates Foundation has made contributions towards vaccine development Global health R&D funding and large-scale disease eradication including malaria and NTDs initiatives.

#### 4.1.4. EML, AMR, and new drug approvals.

New antibacterial agents and artemisinin-based combination therapies for malaria were added to the 2023 WHO Model List of Essential Medicines, and WHO continued to manifest concern towards antimicrobial stewardship [1]. These initiatives are essential but require further global investment and sustainable funding mechanisms to ensure long-term success.

Recent drug approvals have focused on NTDs [24] and AMR [25]. For example, Oral Amphotericin B, which has been developed under UBC’s Global Access Principles, has been listed as a WHO priority medicine for visceral leishmaniasis owing to its cost-effectiveness, thermal stability, and safety in the LMICs [26]. Furthermore, Hancock Lab’s immunomodulatory peptides are effective in combating bacterial, viral, and fungal infections thus supporting WHO’s AMR strategy [26].

### 4.2. Global health strategies and country-specific examples

WHO functions as the primary organization that develops global strategies to combat NTDs. The effectiveness of these strategies depends heavily on how countries execute them. The National Strategic Plan for malaria elimination in India has achieved a substantial reduction in transmission by using MDA campaigns and enhancing diagnostic testing capabilities [27]. The WHO supports Uganda’s MDA program for river blindness, which has effectively decreased the disease prevalence in affected areas [21]. Brazil implemented WHO-recommended Chagas disease control measures for diagnosis treatment and vector control, which led to a decrease in new infections [15]. The WHO collaborated with Senegal to achieve near-eradication of guinea worm disease, which serves as an exemplary model for NTD control [28].

## 5. Recommendations for improving access

Here, we identify and highlight key policy, financial, and technological innovations that fit into global health strategies and have been endorsed by WHO [21], DNDi [29], Gavi [30], and other international organizations that can help achieve the vision of increasing access to essential medicines for NTDs. In support of and advocacy for these approaches, and consistent with the mission of NGDI, we stress the importance of more partnerships and implementation efforts.

Emphasizing the development of domestic pharmaceutical manufacturing in LMICs is to address dependence on imported medicines and increase the affordability of the former. WHO recommends that countries should:

- Invest in the local production of facilities for essential medicines to ensure the availability of a supply chain of NTD treatment.
- Foster regulatory harmonization to streamline the approval process and encourage local drug innovation [27].
- Establish PPPs with international pharmaceutical companies to transfer manufacturing technologies to LMICs. One successful example is the Serum Institute of India, which has recently become the largest producer of affordable vaccines in the world.

### 5.1. Innovative financing mechanisms

- Global health financing: Increased investment from the WHO, World Bank, and Gavi are needed to improve medicine procurement systems, supply chains, and local manufacturing in the LMICs. The Global Fund to Fight AIDS, Tuberculosis, and Malaria provided $4 billion each year to fund medicine access & health system strengthening and research into cost-effective treatment delivery models [31].
- Tiered pricing models: WHO recommends price discrimination on drugs, where poor countries can buy the medicines at reduced prices, while rich countries pay the market price. This strategy has made HIV/AIDS medications available in Africa [22]. However, **tiered pricing may be undermined by parallel trade, where lower-priced medicines are resold in higher-income markets, reducing pharmaceutical companies’ incentives to offer discounted rates.

#### 5.1.1. Expanding public–private partnerships (PPPs).

While existing PPPs have played a role in NTD drug donations, expanding these collaborations requires additional measures:

- Collaboration with pharmaceutical companies: GSK, Novartis, and WHO have engaged in long-term donation programs to fight NTDs, which include donating more than one billion doses of ivermectin for onchocerciasis and praziquantel for schistosomiasis [7].
- R&D incentives for neglected diseases: Governments can introduce tax incentives (e.g., R&D tax credits, reduced corporate tax rates for NTD-related research), grants, and government-assured funding for pharmaceutical companies engaged in the research of NTD drugs.

### 5.2. Leveraging digital health and AI

- AI-driven supply chain management: AI models may help in the forecasting of the demand for medicines and avoiding stockouts as well as in the design of the distribution networks in the LMICs [17].
- Mobile health (mHealth) platforms: Telemedicine and mobile applications can enhance access to clinical services to rural and underserved areas, driving demand awareness for NTD treatments; patients can consult pharmacists, telehealth providers, and medicine delivery services. For example, national health programs to provide remote patient monitoring, chronic disease management, and pandemic response efforts, demonstrating the potential of m Health driven healthcare expansion [26], have utilized the WelTel platform in Rwanda.

## 6. Discussion and conclusion

WHO’s 2023 Essential Medicines List has been updated to address global health needs in 2025, but significant barriers still hinder equitable access to essential medicines for NTDs. To overcome these challenges, we need stronger global and national policy interventions, more funding for R&D, and technology-based solutions. Through improved cooperation and coordination between governments, pharmaceutical companies, and international health organizations, the global health community can enhance availability of essential medicines and work towards the eradication of NTDs.
